# CD5-CK2 Signaling Modulates Erk Activation and Thymocyte Survival

**DOI:** 10.1371/journal.pone.0168155

**Published:** 2016-12-28

**Authors:** Carlos A. Mier-Aguilar, Kevin S. Cashman, Chander Raman, Gloria Soldevila

**Affiliations:** 1 Departamento de Inmunología, Instituto de Investigaciones Biomédicas, Universidad Nacional Autónoma de México, Ciudad de México, México; 2 Division of Clinical Immunology and Rheumatology, Department of Microbiology, University of Alabama at Birmingham, Birmingham, AL, United States of America; 3 Division of Clinical Immunology and Rheumatology, Department of Medicine, University of Alabama at Birmingham, Birmingham, AL, United States of America; University of Alberta, CANADA

## Abstract

CD5 is well recognized for its importance in thymic selection. Although this property of CD5 has been attributed to its ITIM-domain dependent regulation of TCR-signal strength, the mechanism has not been established. A second major signaling domain within the cytoplasmic tail of CD5 is a CK2 binding/activation domain (CD5-CK2BD). Using a gene-targeted mouse in which the CD5-CK2BD is selectively ablated (CD5-ΔCK2BD), we determined that loss of function of CD5-CK2 signaling in a MHC-II selecting TCR transgenic (OT-II) mouse resulted in decrease in double positive (DP) thymocytes, which correlated with enhanced apoptosis. Remarkably, DP cells expressing high levels of CD5 and CD69 and single positive (CD4+SP) thymocytes were increased in CD5-ΔCK2BD mice indicating that CD5-CK2 signaling regulates positive selection and promotes survival. Consistent with this possibility, we determined that the activation and nuclear localization of ERK as well as apoptosis was greater in thymic populations from OTII CD5-ΔCK2BD mice than OTII CD5-WT mice following injection of OVA_323-339_-peptide. The mobilization of Ca^2+^, an early event of TCR activation, was not altered by the loss of CD5-CK2 signaling. Collectively, these data demonstrate that the CD5-CK2 signaling axis regulates positive selection by modulating activation of ERK and promoting survival independent of proximal TCR signals.

## Introduction

T cell differentiation in the thymus is a developmental process tightly regulated by key selection checkpoints, which ensures the generation of a diverse self-restricted and autotolerant T cell repertoire. During this process, TCR mediated signaling has a central role in the final outcome of a functional T cell repertoire, whereby recognition of self-MHC/peptides complexes with low affinity/avidity rescues developing T cells from apoptosis by positive selection, while the absence of recognition of self-ligands leads to death by neglect [1]. Alternatively, recognition of self-ligands with high avidity promotes death by apoptosis through the process of negative selection or rearrangement of the α chain locus to express a different TCR and enter a new selection process [2]. Finally, positively selected thymocytes undergo lineage commitment to become CD4+SP (Single Positive) or CD8+SP mature thymocytes by a process that depends on TCR and co-receptor signaling, although the molecular mechanisms underlying this process still remain controversial (reviewed in [3]). In addition, the thymus also provides the environment required for the selection of self-reactive cells including nTregs, CD8αα+IEL and NKTs under conditions of high avidity (reviewed in [4])

The TCR signaling threshold during thymocyte development is mainly regulated by CD5 [5]. This 67 KDa glycoprotein is expressed on the surface of thymocytes from the double negative (DN) stage, and is upregulated upon pre-TCR signaling and positive selection [6]. Indeed, CD5 expression has been used as a marker for post-selected thymocytes and correlates with the intensity of TCR-mediated signaling during self-peptide/MHC recognition at the DP stage (reviewed in [4]). Several signaling domains within the cytoplasmic tail of CD5 have been proposed to mediate its role as negative regulator of TCR signaling. Among them, an (ITIM)-like motif (pseudo-ITIM) comprising tyrosines 429–441 [7], as well as the carboxy-terminal region containing Y463 [8].

CD5, in addition to regulating TCR signaling, also plays a crucial role in protecting developing thymocytes from apoptosis by a mechanism that involves Akt phosphorylation [9]. Moreover, CK2 a prominent serine/threonine kinase that acts as a major modulator of cell cycle progression and survival, is constitutively associated to S459-S461 residues within the carboxy-terminal region of CD5. We previously demonstrated that the selective absence of CD5-CK2 signaling recapitulated the enhanced AICD in autoreactive T cells and lower EAE severity observed in CD5^-/-^ mice and thereby establishing the relevance of CD5 engaging CK2 to regulate apoptosis [9]. In addition, we recently showed that CD5-CK2 signaling regulates the threshold of T cell activation and T cell differentiation, demonstrating a role for the CD5-CK2 axis in TCR signaling [10].

CK2 constitutively associates with the 458-SSDSD-462 motif within the carboxy-terminal region of CD5 and following CD5 ± CD3 crosslinking becomes activated and phosphorylates CD5 cytoplasmic Ser 459 and 461. Very little is known regarding the CK2 downstream effectors involved in CD5 signaling. In this context, we recently showed that peripheral T cells require CK2 binding to CD5 for optimal Akt activity [11], however these studies were not performed in thymocytes. Therefore, here we sought to determine the role of CD5-CK2 signaling during thymocyte development using a CD5 knock-in mouse in which the nucleotides corresponding to the CK2 binding domain (S458-S461) were deleted.

Our data show that CK2 binding to the CD5 cytoplasmic tail has a specific role during thymocyte selection to negatively regulate ERK phosphorylation and rescue thymocytes from apoptosis independently of Akt phosphorylation. TCR proximal signaling pathways, such as activation of Zap70 and calcium mobilization, were not affected by the absence of CD5-CK2 signaling. From our data, we infer that CD5 in addition to being a negative regulator of TCR signaling, controls pERK levels through the association and activation of CK2, to modulate the process of T cell selection.

## Materials and Methods

### Mice

C57BL/6 mice (CD5-WT) were purchased from the National Cancer Institute Frederick Cancer Research and bred in our colony. C57BL/6 CD5-ΔCK2BD mice were previously described [10]. The OTII TCR^OVA^ (OTII CD5WT) transgenic mice have been previously described [12]. 8–10 week-old mice were used in this study. Additionally, CD5WT and CD5-ΔCK2BD newborn mice were used at the age of 0-24h. All animals were housed and treated in accordance with National Institutes of Health and University of Alabama at Birmingham Institutional Animal Care and Use Committee guidelines. This study was approved by the Institutional Animal Care and Use Committee (IACUC) at University of Alabama"

### Flow cytometry

Staining was performed on thymic and peripheral lymphoid populations after live/dead staining with fixable viability dye eFluor 780 (eBioscience, San Diego, CA). To characterize distinct cellular subpopulations the following Abs were used: anti-CD4 Pacific Blue (RM4-5), anti-CD8 Brilliant Violet 650 (53–6.7, anti-CD69 PE (H1.2F3), anti-TCRVα2 AF 647 (B20.1) and Streptavidin Brilliant Violet 605 all from Biolegend (San Diego, CA); and anti-CD5 PE-Cy7 (53–7.3), anti-CD25 PerCP-Cy5.5 (PC61.5), anti-CD44 FITC (IM7), anti-CD62L bn (MEL-14), anti-CD178 (MFL3) from eBioscience. The Foxp3 intranuclear staining was performed as stated in the eBioscience Foxp3 kit. The following antibodies were utilized to characterize the different thymocyte populations: anti-CD4 Alexa 647 (GK1.5), anti-CD8 PerCP (53–6.7), anti-CD25 FITC (PC61), all from Biolegend, and anti-FoxP3 PE (FJK-16s) from eBioscience. Analysis of phospho-p44/42 MAPK (pERK, Thr202/Tyr204) (D13.14.4E, Cell Signaling), phospho-CDC37 (Ser13; Cell Signaling), phospho-Akt (Ser473; D9E, Cell Signaling), phospho-SAPK/JNK (Thr183/Tyr185, Cell Signaling), phospho Zap-70/Syk (Tyr 493/Tyr526, Cell Signaling), phospho p38 MAPK (T180/Y182) (Clone 30/p38, BD Transduction Laboratories) and Bcl-2 (10C4, eBioscience) was performed as described previously [10]. All samples were collected using either an LSRII flow cytometer (BD Biosciences) or a FACSCalibur cytometer (BD Biosciences) and analyzed using FlowJo software (Treestar®).

### *In vitro* and *in vivo* apoptosis assay

The cells obtained from the thymus of C57BL/6 CD5-WT and the C57BL/6 CD5-ΔCK2BD mice were plated to 96-well plates to a density of 200 x 10^3^ cells in 200 μL. Thymocytes were stimulated for 24h by antibody crosslinking with anti-CD3 (145-2C11, 1 μg/mL) and anti-CD5 (53–7.3, 5 μg/mL). Apoptosis was assessed with Annexin/7-AAD staining kit (Biolegend) following the manufacturer’s protocol. *In vivo* activation induced cell death (AICD) assay of T lymphocytes was performed by injecting i.p. OTII CD5WT and OTII CD5ΔCK2-BD with 300 μg OVA peptide (323–339 ISQAVHAAHAEINEAGR, OVAp; CPC Scientific). After 24 hours, the mice were sacrificed and thymus and spleen were obtained for detection of caspase 3 (eBioscience) in gated populations as previously described [9].

### Imaging flow cytometry

IMAGESTREAM technology was used to analyze intranuclear localization of pERK. Cells were stained with pERK and Hoechst as for flow cytometry. 10,000 cells were acquired using a MARKII Amnis Imagestream (EMD Millipore, MA). For quantitative analysis, between 900 and 1500 pictures were taken of each thymocyte subpopulation. By creating appropriate masks we differentially quantitated cytoplasmic versus nuclear ERK as well as the ratio of nuclear to total pERK, as shown in the representative histograms. IDEAS software was used to analyze the obtained data.

### Calcium mobilization assay

Calcium mobilization in thymocyte suspensions from CD5-WT and CD5-ΔCK2BD mice were performed as described previously [10]. Briefly, thymocytes were stained for CD4 and CD8 and loaded with fluo-4 AM. Ca mobilization in CD4 and/or CD8 gated cells, maintained at 37°C using a specially designed water-jacket, was determined using a BD-LSRII flow cytometer.

### Statistical Analysis

Results were analyzed for statistical significance using unpaired two-tailed Student *t* test using the Graph Pad Software (* p<0.05, ** p<0.01, *** p<0.001, **** p<0.0001).

## Results

### Ablation of CD5-CK2 signaling alters thymocyte development and promotes CD4+ T cell selection

**Fig 1:**
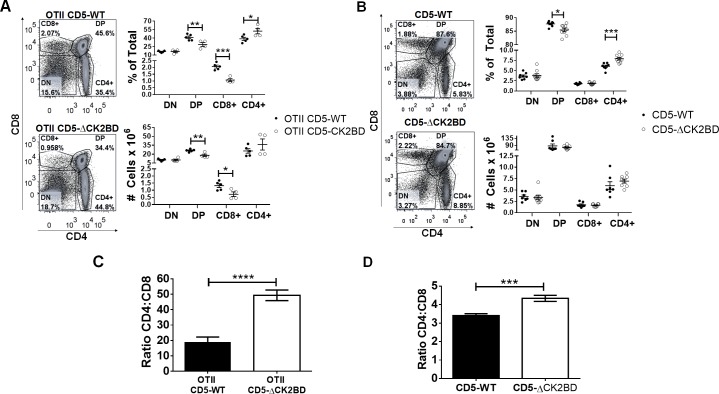
Ablation of CD5-CK2 binding domain alters thymic T cell development. Thymic DN, DP (CD4+CD8+) and SP (CD4+ or CD8+) populations (dot plots), proportions and absolute numbers (graphs) in CD5-WT OTII and CD5-ΔCK2BD OTII TCR-Tg mice (A) and CD5-WT and CD5-ΔCK2BD mice (B). Ratio of CD4:CD8 SP cells in CD5-WT OTII and CD5-ΔCK2BD OTII TCR-Tg mice (C) and CD5-WT and CD5-ΔCK2BD non-TCR Tg mice (D). The flow cytometric dot plots are representative of one of five mice from each strain. The graphs represent data from 4 independent experiments (n = 4–7 each group) *p<0.05, **p<0.01, ***p<0.001, unpaired two-tailed Student-t test.

We examined the contribution of CD5-CK2 signaling in thymic development by breeding the CD5-ΔCK2BD mouse with the TCR^OVA^ transgenic (OTII) mouse. As we previously showed, the inability of CD5 to engage CK2 did not alter the expression of CD5 on any of the thymic T cell populations in TCR transgenic or non-transgenic mice (S1A and S1B Fig). A previous study showed that in contrast to CD5-WT mice, thymocytes in CD5^-/-^ mice had a greater proportion of transgenic TCRβ associating with endogenous TCRα [13]. Here we show that the selective loss of CD5-CK2 signaling did not increase the expression of endogenous Vα or the expression of transgenic Vα2 on all thymic populations (S1C Fig). The lack of CD5-CK2 signaling also had no significant effect on total DN cell numbers or DN subpopulations in OTII TCR-Tg mice or non-transgenic mice (S2 Fig). However, we did find that both proportion and absolute cell numbers of DP thymocytes were significantly lower in CD5-ΔCK2BD compared to CD5-WT OTII TCR-Tg mice (Fig 1A). This contribution of CD5-CK2 signaling on DP cells was also observed in non TCR-Tg mice however, only the differences in proportion were significant (Fig 1B). In OTII TCR-Tg mice the lack of CD5-CK2 signaling resulted in greater frequency and numbers of CD4+ SP T cells and significantly fewer CD8+ SP T cells (Fig 1A). This increase in frequency of CD4+ SP T cells was also observed in CD5-ΔCK2BD non TCR-Tg mice with no change in CD8+ SP T cells (Fig 1B). The data suggest that CD5-CK2 signaling regulates CD4+ T cell selection. Further analysis of the CD4:CD8 ratio showed that in the absence of CD5-CK2 signaling there was a 3-fold increase in the proportion of CD4 versus CD8 cells in the TCR transgenic background (Fig 1C) and about 1.5-fold in the polyclonal background (Fig 1D).

### CD5-CK2 signaling regulates positive selection and maturation of post-selected thymocytes without altering the generation of nTregs.

**Fig 2:**
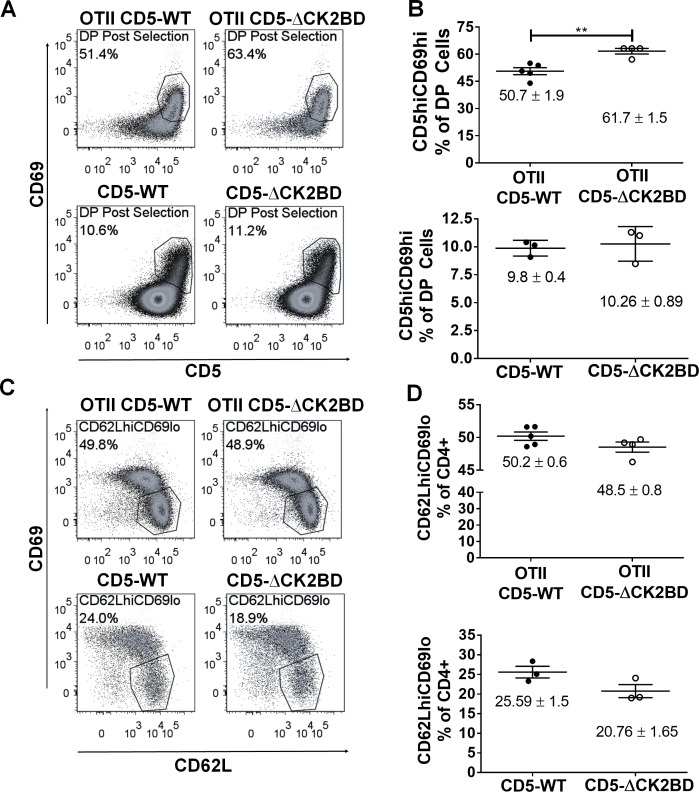
CD5-CK2 signaling regulates positive selection and maturation of post-selected thymocytes. (A) Dot plots of CD5 and CD69 expression gated on DP cells from CD5-WT and CD5-ΔCK2BD OTII TCR-Tg (upper) and non-transgenic (lower) mice. Inset polygon represents the CD5^hi^CD69^hi^ post-selected thymocytes within the DP population. (B) Scatter plot shows the frequency of DP CD5^hi^CD69^hi^ population from CD5-WT and CD5-ΔCK2BD OTII TCR transgenic (upper) and non-transgenic (lower) mice. Each dot represents an individual mouse. (C and D) Fewer terminally differentiated CD4+ SP (CD62L^hi^CD69^lo^) cells in CD5-ΔCK2BD mice. (C) Representative dot plot showing expression of CD69 and CD62L in CD4+SP gated cells from CD5-WT and CD5-ΔCK2BD non-TCR transgenic (upper) and OTII TCR transgenic (lower) mice. The inset polygon represents CD62L^hi^CD69^lo^ terminally differentiated cells. (D) Scatter plot of proportion of CD4+ SP cells with CD62L^hi^CD69^lo^ phenotype from different strains as designated. Each dot represents a mouse. Data represents 4 independent experiments (n = 3–5 mice each group). Numbers are presented as mean± SEM. **p<0.01, unpaired two-tailed Student-t test.

The strength of TCR signaling at the DP stage sets the threshold for positive/negative selection and nTreg generation (reviewed in [14]). We therefore investigated whether the reduction in DP thymocytes in the absence of CD5-CK2 signaling was the result of increased positive selection. To address this question, we analyzed the expression of CD5 and CD69 on the surface of the DP subpopulation, which identifies post-selected thymocytes [15]. The analysis of CD5-ΔCK2BD OTII thymocytes showed a significant increase in the frequency of the DP post-selected subpopulation (CD69^hi^ CD5^hi^) (Fig 2A) compared to CD5 WT OTII although numbers were not affected (S3A Fig). As a final step of maturation, CD4+SP thymocytes down modulate CD69 to acquire competence for emigration from the thymus and home to the peripheral lymphoid organs. To characterize this stage of development, also known as recent thymic emigrants (RTE), we evaluated the expression of CD62L and CD69 on CD4+ SP T cells [16]. We observed a trend towards a decrease in the frequency of CD4+CD62L^hi^CD69^lo^ T cells in CD5-ΔCK2BD OTII TCR-Tg mice compared to CD5 WT OTII TCR-Tg and (Fig 2C and 2D, upper graphs, p = 0.1). Similarly, a decrease in this mature CD4+SP subpopulation was observed in non-transgenic CD5-ΔCK2BD mice compared to CD5WT mice (Fig 2C and 2D, bottom graphs). The results suggest an increase in CD4+ T cell export or a shift in CD4+ maturation or survival. In this context, increased levels of CD69 were observed on CD5-ΔCK2BD CD4+SP thymocytes, indicating a defect in final CD69 downregulation, which is a prerequisite for thymic export (S3C Fig).

**Fig 3:**
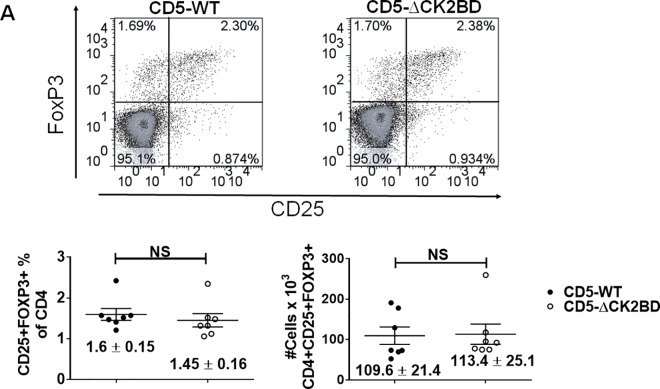
Generation of nTregs is not affected by the absence of CD5-CK2 binding domain. (A) Dot plots (upper) of CD25 and FoxP3 expressing cells within CD4 gated cells. Scatter plots showing proportion and total cell numbers of CD4+CD25+FoxP3+ (lower). Each dot represents one mouse. Data represents 3 independent experiments (n = 7 mice each group). Numbers are presented as mean ± SEM; NS = not significant, unpaired two-tailed Student-t test.

Within CD4+SP thymocytes, high TCR avidity selects for nTreg cells and this population is expanded in mice lacking CD5 [9]. In peripheral T cells, CD5-CK2 signaling modulates Akt activity [11], a signaling pathway down regulated during the generation of nTreg [17, 18]. We observed no difference in frequency or numbers of nTreg cells (CD4+CD25+Foxp3+) between CD5-CK2ΔBD and CD5 WT mice (Fig 3A and 3B). This result suggests that the CD5-CK2 signaling axis is not involved nTreg selection in the thymus and therefore is not responsible for the increased CD4:CD8 ratio observed in CD5-CK2ΔBD mice; however, other domains within the cytoplasmic tail of CD5 shown to regulate TCR signaling, such as the ITIM (Y429-441) domain or the carboxy-terminal Y463, may regulate nTreg generation.

### Increased *in vitro* and *in vivo* apoptosis in the absence of CD5-CK2 signaling

**Fig 4:**
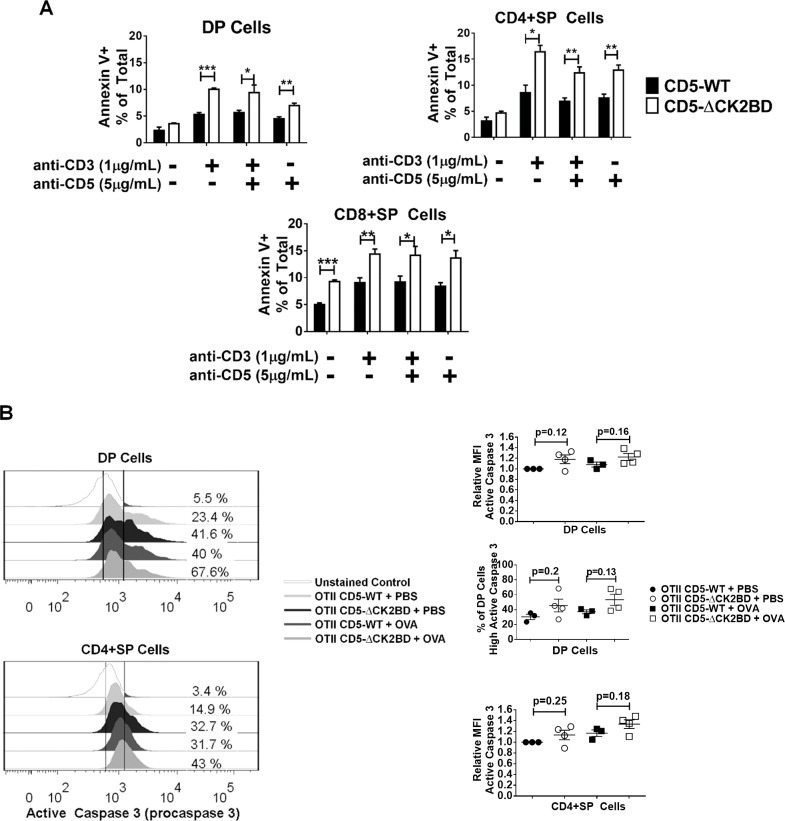
Enhanced *in vitro* and *in vivo* thymocyte apoptosis in the absence of the CD5-CK2BD. (A) Apoptosis (Annexin V^+^ 7-AAD^-/+^) in thymocytes from CD5-WT and CD5-ΔCK2BD mice following culture for 24h in the presence of α-CD3 and/or α-CD5 or medium alone. The graphs show apoptosis in gated DP, CD4+SP and CD8+SP and populations. Data represent mean±SEM from one experiment (n = 3–4 independent mice). (B) Histograms (lower left) show proportion of cells with elevated caspase 3 levels relative to unstained cells within DP and CD4+SP thymocytes obtained from CD5-WT and CD5-ΔCK2BD OTII TCR-Tg mice after i.p injection with either PBS or OVAp. Solid vertical lines are drawn to reflect unstained MFI and to identify the caspase 3 on the highest expressing population. Scatter plots show MFI of caspase 3 levels in DP cells (top), proportion of DP cells expressing high levels of caspase 3 (middle) and MFI of high caspase 3 expressing CD4+ SP cells (bottom). Data represents 4 independent experiments (n = 4 mice each group). Numbers are presented as mean±SEM, *p<0.05, unpaired two-tailed Student-t test.

CD5 has been shown to function as a pro-survival receptor in thymocytes (reviewed in [19]). In peripheral T cells, this function is compromised if CD5-CK2 signaling is ablated [10, 20]. Therefore, the lower numbers of DP cells observed in CD5-ΔCK2BD mice may reflect enhanced activation induced cell death (AICD) during selection. We tested for this possibility using *in vitro* and *in vivo* approaches. Thymic T cells from CD5-WT mice and CD5-ΔCK2BD mice were cultured for 24 hours in the presence or absence of anti-CD3 ± anti-CD5 and apoptosis was quantitated using Annexin V and 7-AAD. We found that apoptosis under all stimulation conditions in DP and SP (CD4+ and CD8+) thymocytes obtained from the CD5-ΔCK2BD mice was greater than CD5-WT mice (Fig 4A). Furthermore, thymic populations from CD5-ΔCK2BD in media alone exhibited higher apoptosis, but the difference from CD5-WT thymocytes was significant only for CD8+ SP cells. Apoptosis in DN cells were similar between CD5-WT and CD5-ΔCK2BD (S2 Fig). To test for susceptibility to AICD *in vivo*, we injected OTII TCR-Tg mice with PBS (control) or OVA peptide (300 μg) and measured expression of caspase 3. We found that DP and CD4+ SP thymocytes from CD5-ΔCK2BD OTII TCR-Tg mice had a slight increase in the proportion of cells expressing elevated levels of caspase 3 at basal conditions (PBS injected) and following injection with OVA peptide compared to CD5-WT OTII TCR-Tg mice (Fig 4B). These results show a prominent role of the CD5-CK2 signaling axis in promoting thymocyte survival.

### CD5-CK2 mediated thymocyte survival is independent of Akt and CDC37 activation.

**Fig 5:**
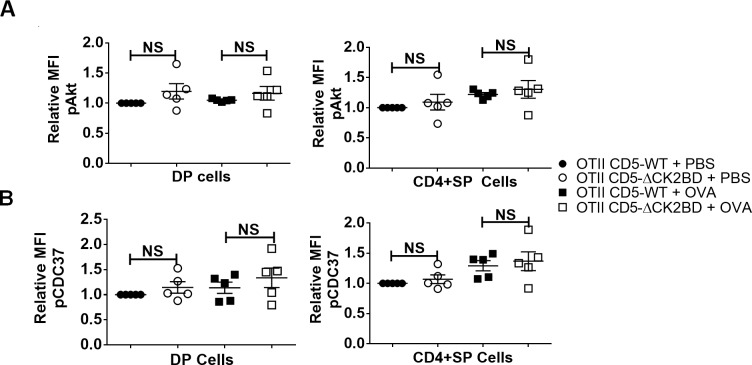
AKT and CDC37 activation is unaffected by loss of CD5-CK2 signaling pathway. Scatter plot of pAKT(S473) levels (A) and pCDC37 (S13) levels (B) within DP or CD4+SP thymocytes obtained from CD5-WT OTII and CD5-ΔCK2BD OTII TCR-Tg mice after injection with PBS (control) or 300 μg of OVAp. Data represents 4 independent experiments (n = 5 mice each group). NS = not significant, unpaired two-tailed Student-t test.

Akt, a substrate of CK2 [21] and a downstream effector of TCR signaling, is a key regulator of cell death in thymocytes [9]. In peripheral T cells, CD5-CK2 signaling is necessary for efficient TCR-induced activation of Akt to promote cell survival [11]. We found no difference in the activation of Akt in DP or SP thymocytes under basal conditions or following OVAp injection between CD5-WT and CD5-ΔCK2BD mice (Fig 5A). CDC37, activated by CK2, functions as a co-chaperone for heat shock protein 90 (HSP90) to promote survival [22]. We found that loss of CD5-CK2 signaling had not effect on OVAp induced pS13-CDC37, the site of CK2 phosphorylation (Fig 5B). These findings suggest pAkt/pCDC37 signaling axis is not regulated via CD5-CK2 signaling in developing T lymphocytes unlike previous findings in peripheral T lymphocytes [11].

### Elevated ERK activation in the absence of CD5-CK2 signaling

**Fig 6:**
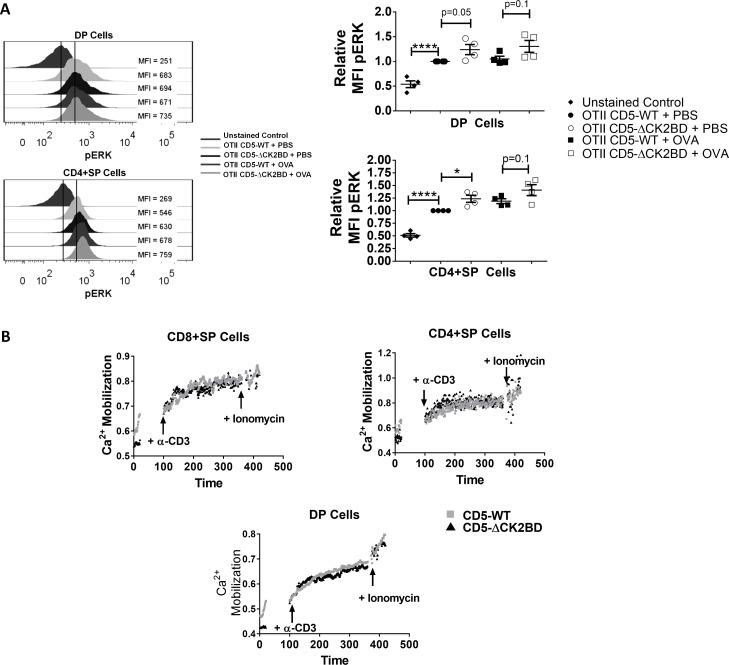
Deletion of the CD5-CK2BD results in increased basal and induced ERK phosphorylation in the absence of enhanced TCR proximal signaling. (A) Histograms (upper left) show pERK levels within DP or CD4+SP thymocytes obtained from CD5-WT OTII and CD5-ΔCK2BD OTII TCR-Tg mice after PBS or OVAp injection. Vertical solid lines are drawn to identify the MFI of unstained cells and the MFI of cells from PBS treated mice. Scatter plots (right) show data from 4 individual mice. (B) Calcium mobilization in DP, CD4+SP and CD8+SP thymocytes from CD5-WT and CD5-ΔCK2BD mice following stimulation with α-CD3. Each graph is representative of 2 independent experiments (n = 2–3 independent mice). Inset arrows denote time point of addition of α-CD3 or ionomycin. *p<0.05, ****p<0.0001, unpaired two-tailed Student-t test.

Among the CK2 downstream effectors, ERK is a protein relevant for TCR signaling and survival, [21]. Sustained pERK levels are required for positive selection of thymocytes and CD4+SP lineage commitment [23], however acute activation of ERK leads to apoptotic death [24]. In CD5-ΔCK2BD mice, DP and CD4+SP cells had higher basal levels of pERK compared to CD5-WT mice that was significantly increased after OVAp injection (Fig 6A). The higher basal pERK correlates with the enhanced positive selection to CD4+SP thymocytes in CD5-ΔCK2BD mice and CD4+SP differentiation, and might also be responsible for the increased cell death observed in CD5-ΔCK2BD thymocytes (Fig 2 and Fig 3A).

To investigate whether increased pERK was the result of enhanced TCR proximal signaling, we evaluated TCR-induced calcium mobilization in thymocytes from CD5-ΔCK2BD mice. The absence of CD5-CK2 signaling did not affect calcium flux (Fig 6B), thus suggesting an alternative CD5-dependent mechanism for the regulation of ERK activation. Overall, the data suggest that CD5-CK2 signaling promotes thymocyte survival by regulating acute activation of ERK.

### Enhanced TCR mediated apoptosis and elevated Erk phosphorylation in CD5-CK2ΔBD is independent of peripheral T cell activation

**Fig 7:**
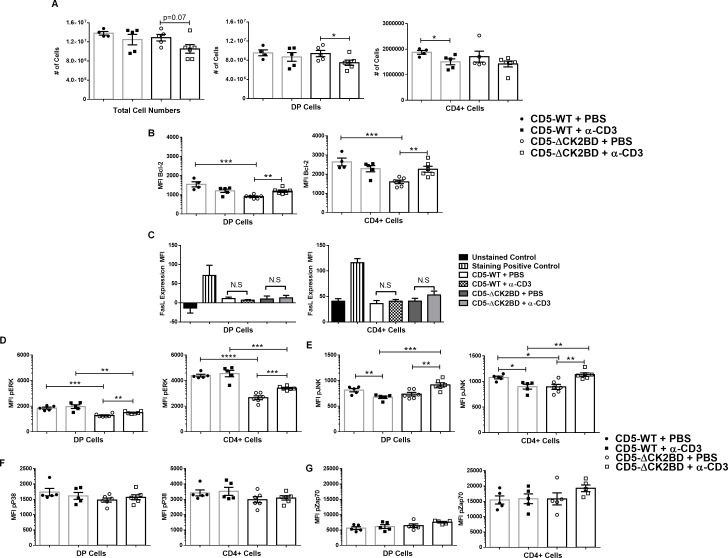
Increased TCR dependent MAPK activation and enhanced AICD in CD5-CK2BD deficient mice is independent of peripheral T cells activation and thymocyte FasL expression. (A) Total cell numbers (left panel) of CD5-WT vs CD5-ΔCK2BD newborn mice after injection of either PBS (control) or α-CD3 (20 μg) for 24h. To evaluate the effect of anti-CD3 stimulation, both CD5-ΔCK2BD or CD5-WT were compared with their respective unstimulated controls. **S**ubpopulation analysis show DP (middle panel) or CD4+ SP thymocytes (right panel) (B) Scatter plots showing Bcl-2 levels in DP and CD4+ SP thymocytes. (C) Bar graphs of FasL expression in PBS injected and anti-CD3 injected mice. Adult thymocytes were used as positive control. (D) Scatter plots show pERK expression 24h after α-CD3 injection or PBS as in DP and CD4+ SP cells from CD5-WT and CD5-ΔCK2BD newborn mice. (E) Graphs showing expression of pJNK (F) pP38 and (G) pZap70. Each graph is representative of 1 independent experiment (n = 5–6 each group). *p<0.05, **p<0.01, ***p<0.001, unpaired two-tailed Student-t test.

Hyperactivation of peripheral T cell can induce/augment thymocyte death [25, 26]. We therefore interrogated if enhanced TCR complex signal-dependent apoptosis and ERK activation in CD5-ΔCK2BD thymocytes was intrinsic to thymic development or an extrinsic effect of peripheral T cell activation. CD5-ΔCK2BD or CD5-WT newborn mice (0-24h after birth) were injected with α-CD3 (20 μg) or PBS i.p. and 24h later thymi were harvested for analysis. Since newborn mice have very few to none mature T cells, injection of TCR agonists do not induce death of thymocytes from secondary factors [25, 27, 28]. Similar to that observed in adult thymus (Fig 4), TCR engagement in CD5-ΔCK2BD resulted in greater loss in numbers of total thymocytes and DP thymocytes than in CD5-WT mice (Fig 7A). In order to elucidate the underlying mechanism for the increased cell death observed in the absence of CD5-CK2 signaling, we analyzed for changes in expression of the anti-apoptotic molecule, Bcl-2 and AICD associated molecules, FasL (Fig 7) and Fas (not shown). We observed reduced basal levels of Bcl-2 in CD5-ΔCK2BD DP and CD4+ SP thymocytes mice compared to WT, however, upon CD3 crosslinking Bcl2 levels increased only in CK2-signaling deficient thymocytes (Fig 7B). Under certain conditions AICD during negative selection in thymus is dependent on Fas-FasL [27, 29]. We observed no upregulation of FasL on any thymocyte population from CD5-WT or CD5-ΔCK2BD mice (Fig 7C). Overall these data argue against an effect caused by extrinsic cell death pathways.

As observed in adult mice, TCR activation led to increased ERK phosphorylation in CD5-ΔCK2BD mice but not in CD5-WT newborn mice (Fig 6 and Fig 7D). However, unlike the observation in adult mice, basal pERK levels were lower in CD5-ΔCK2BD mice compared to CD5-WT mice (Fig 7D). Since peripheral T cells are not activated in newborn mice, these results further demonstrate that CD5-CK2 signaling intrinsically regulates TCR dependent ERK phosphorylation in thymocytes.

Other MAPK, such as JNK and P38, have been implicated in regulating survival versus cell death during thymocyte selection (reviewed in [1]). Although, basal levels of pJNK were significantly lower in CD5-ΔCK2BD CD4+ SP cells, anti-CD3 treatment enhanced pJNK levels in CD5-ΔCK2BD DP and CD4+ SP T cells; in contrast, such *in vivo* stimulation reduced pJNK levels in CD5-WT mice (Fig 7E). In neonatal mice, we observed no difference in pP38 between CD5-WT and CD5-ΔCK2BD mice (Fig 7F).

Finally, TCR-dependent Zap70 phosphorylation was not different between CD5-ΔCK2BD and CD5-WT newborn mice (Fig 7G). This result indicates that TCR proximal signals in newborn mice and adult mice (Fig 6B) do not contribute to the enhanced MAPK activation observed in CD5-ΔCK2BD thymocytes.

### Nuclear pERK localization is not regulated through CD5-CK2 binding domain in developing T lymphocytes.

**Fig 8:**
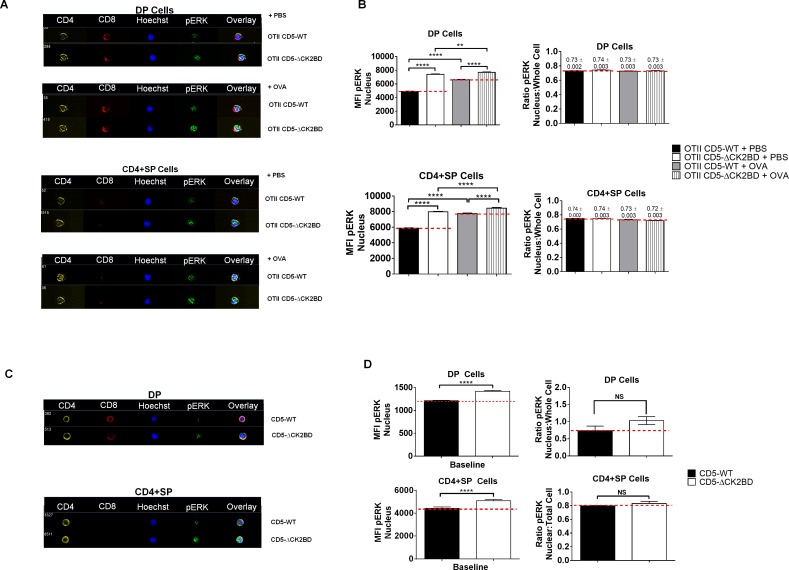
Nuclear translocation pERK is independent of CD5-CK2 signaling. (A) Images of DP and CD4+SP thymocytes obtained from PBS or OVAp injected CD5-WT OTII and CD5-ΔCK2BD OTII TCR-Tg mice and analyzed by imaging flow cytometry. The images show each channel individually represented as well as an overlay of all channels. The data are representative of at least 900 images each from 2 independent experiments (n = 2 mice each group). (B) Quantitative representation of all events represented in (A). The graphs reflect mean±SEM pERK levels inside the nucleus (left panels) or ratio of pERK in nucleus to whole cell (right panels) within DP (upper panels) or CD4+SP (lower). (C) Images of pERK in DP and CD4+SP thymocytes obtained from non-TCR transgenic CD5-WT and CD5-ΔCK2BD mice. The images are representative of 900–1500 images from 3 independent experiments (n = 3 mice each group). (D) Quantitative representation analyzed similar to (B). **p<0.01 ****p<0.0001, unpaired two-tailed Student-t test.

The phosphorylation of ERK by CK2 on two Ser residues promotes translocation into the nucleus where it exerts its function as a modulator of transcriptional activity [30–32]. We used imaging flow cytometry to quantitatively assess whether the subcellular localization of pERK was altered in the absence of CD5-CK2 signaling. Validating the flow cytometry data, we found that the total pERK levels in DP and CD4+ SP thymocytes were higher in CD5-ΔCK2BD mice at basal and following *in vivo* activation using OVAp (S4A Fig). We quantitatively distinguished the levels of pERK in the cytoplasm and nucleus at basal and following OVAp injection to determine if pERK entry to the nucleus was altered in thymocytes from CD5-ΔCK2BD mice. The data showed higher basal (Fig 8A, 8B, 8C and 8D) and OVAp (Fig 8A and 8B) induced pERK levels in the nuclei of DP and SP populations from CD5-ΔCK2BD compared to CD5 WT mice. OVAp injection increased pERK levels in both CD5-WT and CD5-ΔCK2BD OTII-Tg mice, however, the magnitude of increase was greater in CD5-WT thymocytes (Fig 8A and 8B). Remarkably, the ratio between nuclear and total pERK was similar between CD5-ΔCK2BD and CD5-WT mice (Fig 6E). This indicates that CD5 dependent CK2 activation is not involved in ERK translocation into the nucleus.

## Discussion

We previously reported that ablation of the ability of CD5 to engage and activate CK2 in peripheral T cells attenuated EAE disease severity by enhancing T cell death and hampering differentiation to encephalitogenic Th17 cells [10, 20]. However, we did not investigate if CD5-CK2 signaling played a role in thymocyte development. In this study we report that in CD5-ΔCK2BD mice the proportion of CD5^hi^CD69^hi^ DP and CD4 SP thymocytes is increased showing that the CD5-CK2 signaling pathway regulates positive selection. The increase in CD4+SP thymocytes in CD5-ΔCK2BD mice indicated an enhanced CD4+ T cell selection, as demonstrated by the significant increase in the CD4:CD8 ratio. Our results provide a potential mechanism for previous findings that showed expanded numbers of transitional CD4+CD8^int^ thymocyte population in CD5^-/-^ MHC Class II^-/-^ mice [33]. Thus, the increased CD4:CD8 ratio observed in the absence of CD5-CK2 signaling might be the result of enhanced TCR mediated signals. Positive selection and CD4+ lineage commitment requires sustained ERK activation [23]. Therefore, the increased ERK phosphorylation observed in CD5-ΔCK2BD mice supports the role for CD5-CK2 signaling in down regulation of positive selection [34] and in restraining CD4+SP selection. This is in agreement with the signal strength model of CD4 versus CD8 commitment (reviewed in [3]). Alternatively, the basal enhanced cell death CD8+ SP CD5-ΔCK2BD thymocytes might reflect the proposed model of ‘asymmetric’ apoptosis between CD8+SP and CD4+SP thymocytes [35].

TCR crosslinking in CD5^-/-^ T cells resulted in increased calcium mobilization and hyperphosphorylation of TCRζ, ZAP70, PLC-γ[13] and ERK [9, 36]. In contrast, CD5-CK2 signaling deficient T cells have normal TCR-induced calcium flux and activation of Zap70, arguing in favor of an alternative CD5-CK2-dependent pathway in downregulation of ERK phosphorylation, independent of proximal TCR signaling. In this context, CK2α has been shown to interact with PP2A, a negative regulator of ERK signaling [37], which was also shown to co-immunoprecipitate with CD5 in human Jurkat T cells [38].

The maturation and emigration of post-selected SP thymocytes requires cessation of TCR signaling and downregulation of CD69 expression (reviewed in [39]). CD69 expression is upregulated by AP-1 a nuclear downstream target of ERK [40]. Therefore, the reduced proportion of CD4^+^CD62L^hi^CD69^lo^ thymocytes observed in CD5-ΔCK2BD mice is consistent with the sustained levels of elevated ERK phosphorylation, correlating with increased surface levels of CD69 within CD4+SP subpopulation. An alternative hypothesis to explain for fewer CD69^lo^ CD4+ post-selected CD5-ΔCK2BD thymocytes is enhanced thymic export; however, our previous results found that CD4+ population in peripheral lymph nodes is reduced in the absence of CD5-CK2 signaling [10]. Overall our data support a requirement for CD5-CK2 signaling for efficient maturation of post-selected thymocytes.

CD5 expression strongly correlates with the strength of TCR signaling [6]. Interestingly, the lack of CD5-CK2 signaling had no effect on expression of CD5 on any thymic subpopulation Thus, TCR-induced CD5 upregulation is likely to be regulated by other CD5 cytoplasmic regions, like the CD5-ITIM domain [7]. The elevated basal pERK observed in adult mice led us to predict that nTreg numbers would be expanded in CD5-ΔCK2BD thymus, as we previously showed in CD5^-/-^ mice [9]. Several TCR proximal downstream effector are required for optimal nTreg generation, including PLCγ1 [41], LAT [42], arguing in favor of the need of a strong signal threshold for their selection (reviewed in [43]). However, we found no expansion of nTreg cells in the absence of CD5-CK2 signaling indicating that other than ERK, proximal TCR signals, unaffected in CD5-ΔCK2BD mice, are involved in nTreg generation. Additionally, inhibition of the Akt/mTOR axis is required during nTreg selection [17], which was unaltered in CD5-ΔCK2BD mice. Finally, although CK2 is highly expressed in nTregs compared to effector T cells, our results show that CK2 associated with CD5 is not relevant for nTreg selection [44].

CD5 has been described as a pro-survival receptor in T cells (reviewed in [19]). Among the possible molecules involved in CD5-mediated survival, CK2 [10, 20], Akt [9] and ERK [45] are postulated to play a role in this process. CDC37 and Akt are two major intracellular proteins shown to be crucial for CK2 mediated survival [21, 46]. However, the phosphorylation of these molecules was not altered in the absence of CD5-CK2BD, suggesting that other CD5 dependent CK2 downstream effectors are involved. In thymus, unlike in peripheral T cells, the efficient activation Akt, is independent of CD5-CK2 signaling [11]. While sustained activation of ERK is necessary for thymocyte survival and positive selection, acute activation of ERK activation such as during negative selection results in cell death [23].

AICD of DP thymocytes can be mediated by cytokines and/or increased steroid levels released by mature post-thymic T cells when hyper-activated [25, 26]. Peripheral CD4 T cells from CD5-ΔCK2BD mice are hypoactive to antigen stimulation [10]. This in itself indicates that the enhanced AICD in CD5-ΔCK2BD thymocytes is unlikely to be due to peripheral T cell activation. We show here that enhanced TCR/CD3 induced AICD also occurred in CD5-ΔCK2BD newborn mice that do not have significant numbers of mature post-thymic T cells. From this we can infer that CD5-CK2 signaling intrinsically promotes survival in developing thymocytes during negative selection. Analysis of Bcl-2 expression in CD5-ΔCK2BD newborn mice showed that under basal conditions the absence of CD5-CK2 resulted in decreased Bcl-2 levels, while after CD3 stimulation Bcl-2 is enhanced to the levels of WT mice suggesting that under steady state conditions this anti-apoptotic molecule may promote CK2-dependent thymocyte survival, while under AICD conditions other pro- or anti-apoptotic molecules may be involved.

We further showed that this pro-survival activity of CD5-CK2 signal is active in the absence of AICD induced by Fas-FasL, arguing against an effect caused by extrinsic cell death pathways. This is of significance, since the contribution of Fas-FasL in negative selection is dependent on strength of TCR signals [27, 29]. As previously described by Kishimoto and Sprent [27], we found that in WT newborn mice anti-CD3 stimulation induced apoptosis of CD4+ SP but not of DP thymocytes. In contrast, when CD5-CK2 signaling is ablated, we find significant anti-CD3 induced apoptosis in newborn DP thymocytes, rather than CD4+ SP T cells. This might reflect accelerated progression from DP stage to SP stage, in addition to enhanced DP cell death, as suggested in adult mice (Fig 2A and 2B).

Our data showed that CD5-CK2 signaling regulates both sustained and acute activation of ERK, thus having an impact on positive selection and thymocyte survival. Analysis of Erk phosphorylation by Imagestream further confirmed and provided more statistical power to the data obtained with standard flow cytometry (Fig 8 and S4 Fig.). Interestingly, in contrast to adult mice, the absence of CD5-CK2 signaling in neonatal mice, led to reduced basal levels of p-Erk. However, both adult and neonatal thymocytes from CD5-ΔCK2BD mice showed enhanced phosphorylation of Erk in response to CD3, compared to their WT counterparts (Figs 6 and 7). Although, phosphorylation of pERK by CK2 is shown to promote its entry into the nucleus [30], we found that this was not altered in CD5-ΔCK2BD thymocytes. This would indicate that CK2 activated by CD5 engagement does not participate directly in Erk translocation. Additionally, we analyzed JNK and P38 phosphorylation, as these MAPK have also been involved in thymocyte selection (reviewed in ([1]). Similar to ERK, enhanced phosphorylation of JNK, but not of p38, was detected in newborn CD5-ΔCK2BD thymocytes in response to CD3 stimulation. Although P38 has recently been shown to promote thymocyte survival and can be activated downstream CD5 [47], our data suggest that CD5 does not mediate P38 dependent signals during thymocyte development.

Collectively, our data demonstrate a key role of CK2 in CD5-mediated regulation of thymocyte selection and survival, by controlling the phosphorylation of ERK independent of proximal TCR signals. This result also reveals an important function of CK2 in thymocyte selection. We also suggest that the dogma stating that CD5 dependent negative regulation of TCR proximal signals is primarily involved in thymocyte selection, insufficiently addresses the mechanism by which CD5 modulates thymic development. A knock-in mouse in which the ITIM domain of CD5 is selectively mutated will be an important tool to discriminate between CD5-ITIM and CD5-CK2 signaling activities.

## Supporting Information

S1 Fig Surface expression of CD5 and Va2 are not altered in the absence of CD5-CK2 signaling. (A) Histograms representing CD5 levels in the different thymocyte subpopulations of both CD5-WT and CD5-ΔCK2BD non-Tg (A) and OTII transgenic (B) are shown. (C) Histogram representation of Vα2 expression of the different thymocyte subpopulations of CD5-WT OTII and CD5-ΔCK2BD OTII.
(TIF)

S2 Fig DN subpopulations are unaffected in CD5-ΔCK2BD mice. Dot plots showing CD25 and CD44 expression within DN subpopulation (from Fig 1) are shown (left panels). Gates were set to identify DN1, DN2, DN3 and DN4 subpopulations based on CD25 and CD44 expression in OTII transgenic (A) or non-Tg (B) mice in the CD5-WT versus CD5-ΔCK2BD backgrounds. Scatter plot (right panels) represent cell proportions of each thymocyte subpopulation from individual mice. (C) Apoptosis (Annexin V^+^ 7-AAD^-/+^) in thymocytes from DN thymocytes CD5-WT and CD5-ΔCK2BD mice following culture for 24h in the presence of α-CD3 and/or α-CD5 or medium alone. Each graph is representative of 4 independent experiments (n = 4–7 mice) **p<0.01, unpaired two-tailed Student-t test.
(TIF)

S3 Fig Increased positive selection and maturation of post-selected thymocytes in CD5-ΔCK2BD mice. (A) Scatter plot (right panel, CD5-WT and CD5-ΔCK2BD OTII, left panel CD5-WT and CD5-ΔCK2BD non-Tg) representing total cell numbers from Fig 2A and 2B (CD5^hi^CD69^hi^ cells). (B) Scatter plot (right panel, CD5-WT and CD5-ΔCK2BD OTII, left panel CD5-WT and CD5-ΔCK2BD non-Tg) representing total cell numbers from Fig 2C and 2D. (CD4+CD62L^hi^CD69^lo^). Each dot represents an independent mouse. (C) Graph representation of CD69 levels within CD4+SP cells in CD5-WT and CD5-ΔCK2BD non-Tg mice. Data representative of at least 3 independent experiments (n = 3–5 mice). Numbers are presented as mean± SEM. *p<0.05 **p<0.01. NS represents no statistical significance, unpaired two-tailed Student-t test.
(TIF)

S4 Fig Increased nuclear and cytosolic pERK in CD5-ΔCK2BD thymocytes. (A) Graphs representing total pERK levels detected by Imaging Flow Cytometry in CD4+SP and DP cells obtained from CD5-WT OTII and CD5-ΔCK2BD OTII TCR-Tg under basal conditions or after OVA stimulation (left panels), and from CD5-WT and CD5-ΔCK2BD non-Tg mice under basal conditions (right panels). (B) Histograms from a representative experiment, showing pERK levels in whole cell, cytoplasm and nucleus of CD4+SP cells obtained from CD5-WT (B) and CD5-ΔCK2BD non-Tg mice, under basal conditions. Nucleus:Whole cell ratio are also represented. (B and C). Data represent 900–1500 images of 2–3 independent experiments (n = 2–3 mice) ****p<0.0001, unpaired two-tailed Student-t test.
(TIF)
