# Supplementary figures and images for: CD5-CK2 Signaling Modulates Erk Activation and Thymocyte Survival
Source: PLoS One. 2016 Dec 28;11(12):e0168155. doi: 10.1371/journal.pone.0168155 (PMC5193405; doi:10.1371/journal.pone.0168155)

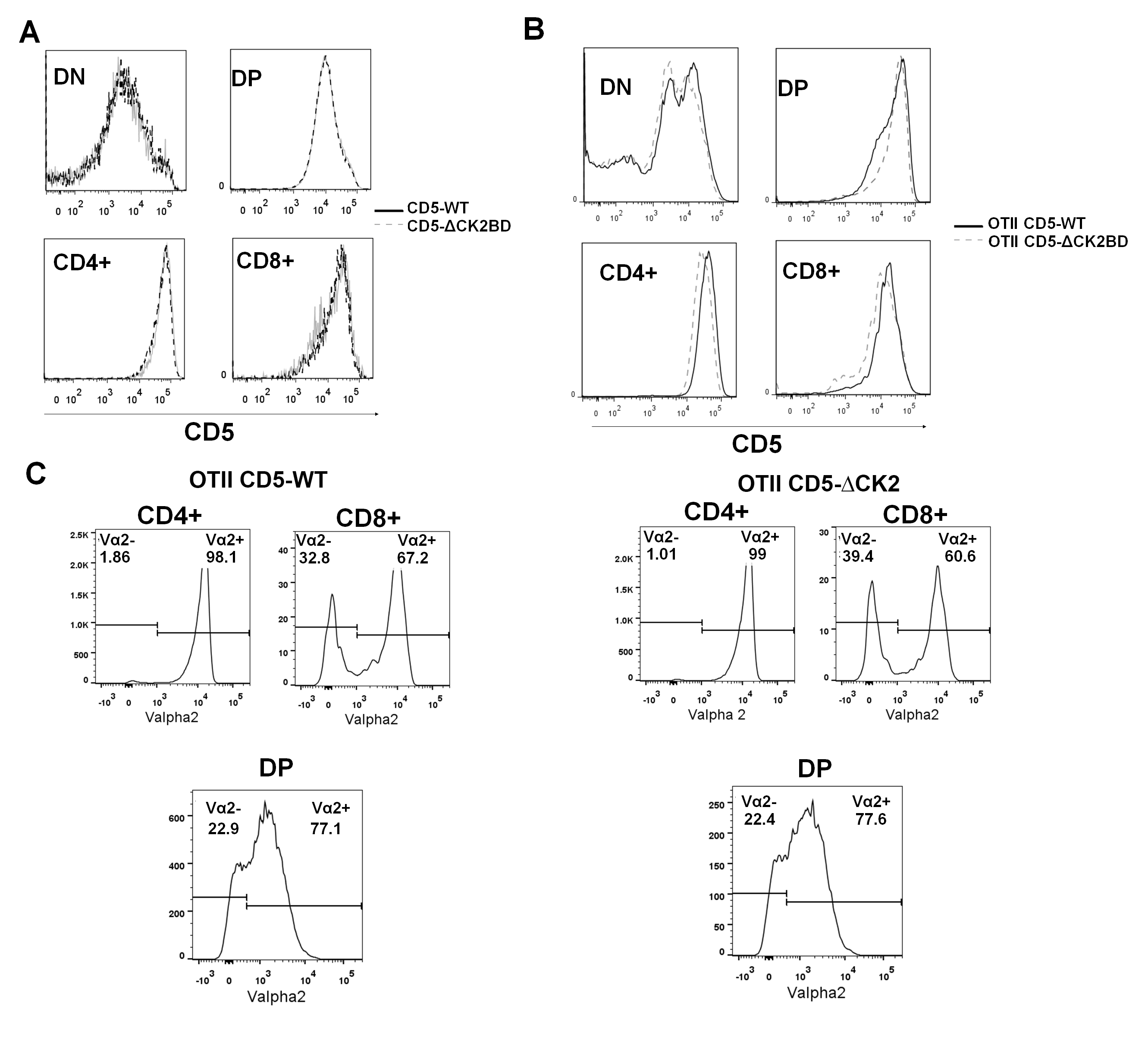

Supplement: S1 Fig — (A) Histograms representing CD5 levels in the different thymocyte subpopulations of both CD5-WT and CD5-ΔCK2BD non-Tg (A) and OTII transgenic (B) are shown. (C) Histogram representation of Vα2 expression of the different thymocyte subpopulations of CD5-WT OTII and CD5-ΔCK2BD OTII. (TIF) [file pone.0168155.s001.tif]

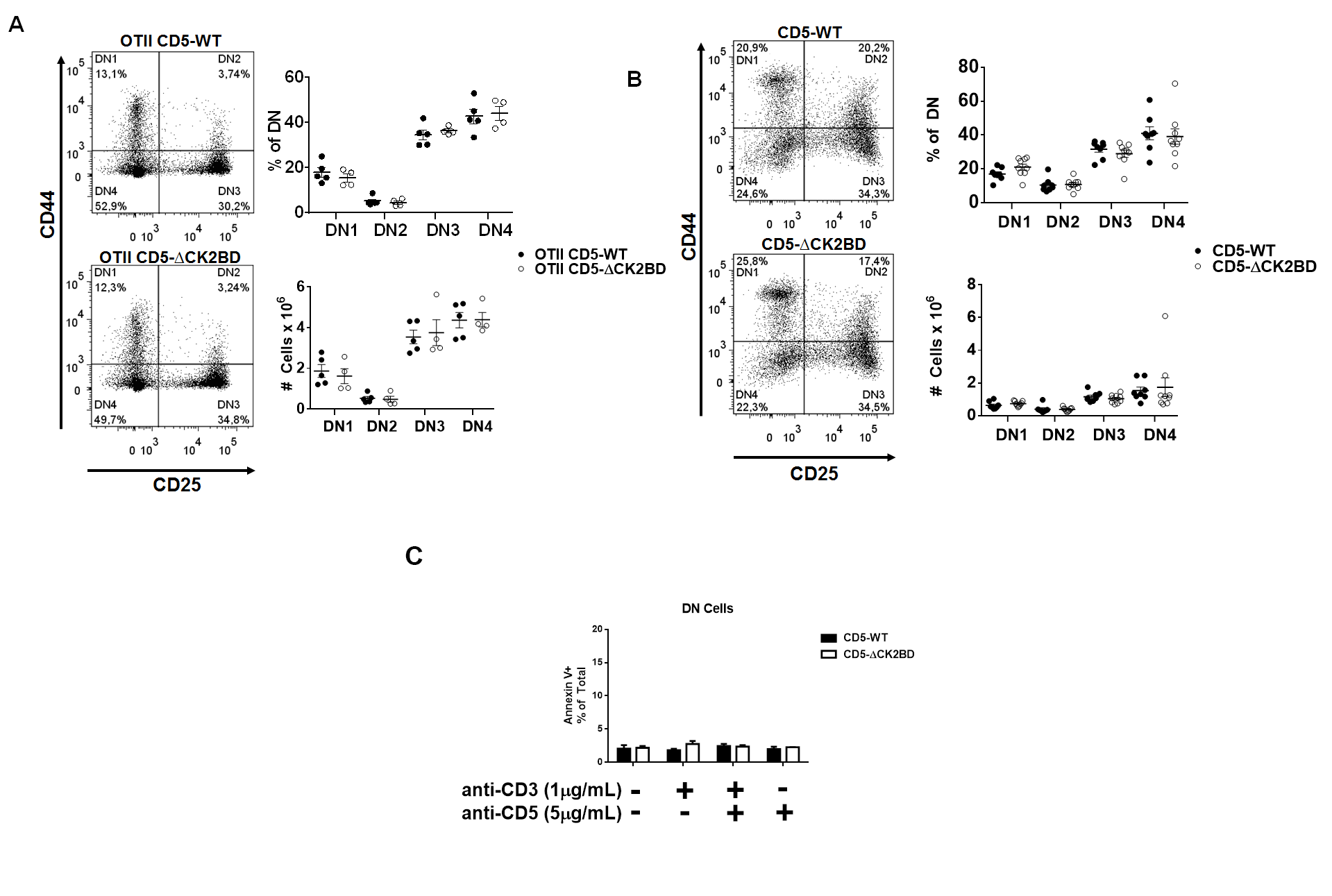

Supplement: S2 Fig — Dot plots showing CD25 and CD44 expression within DN subpopulation (from Fig 1) are shown (left panels). Gates were set to identify DN1, DN2, DN3 and DN4 subpopulations based on CD25 and CD44 expression in OTII transgenic (A) or non-Tg (B) mice in the CD5-WT versus CD5-ΔCK2BD backgrounds. Scatter plot (right panels) represent cell proportions of each thymocyte subpopulation from individual mice. (C) Apoptosis (Annexin V+ 7-AAD-/+) in thymocytes from DN thymocytes CD5-WT and CD5-ΔCK2BD mice following culture for 24h in the presence of α-CD3 and/or α-CD5 or medium alone. Each graph is representative of 4 independent experiments (n = 4–7 mice) **p<0.01, unpaired two-tailed Student-t test. (TIF) [file pone.0168155.s002.tif]

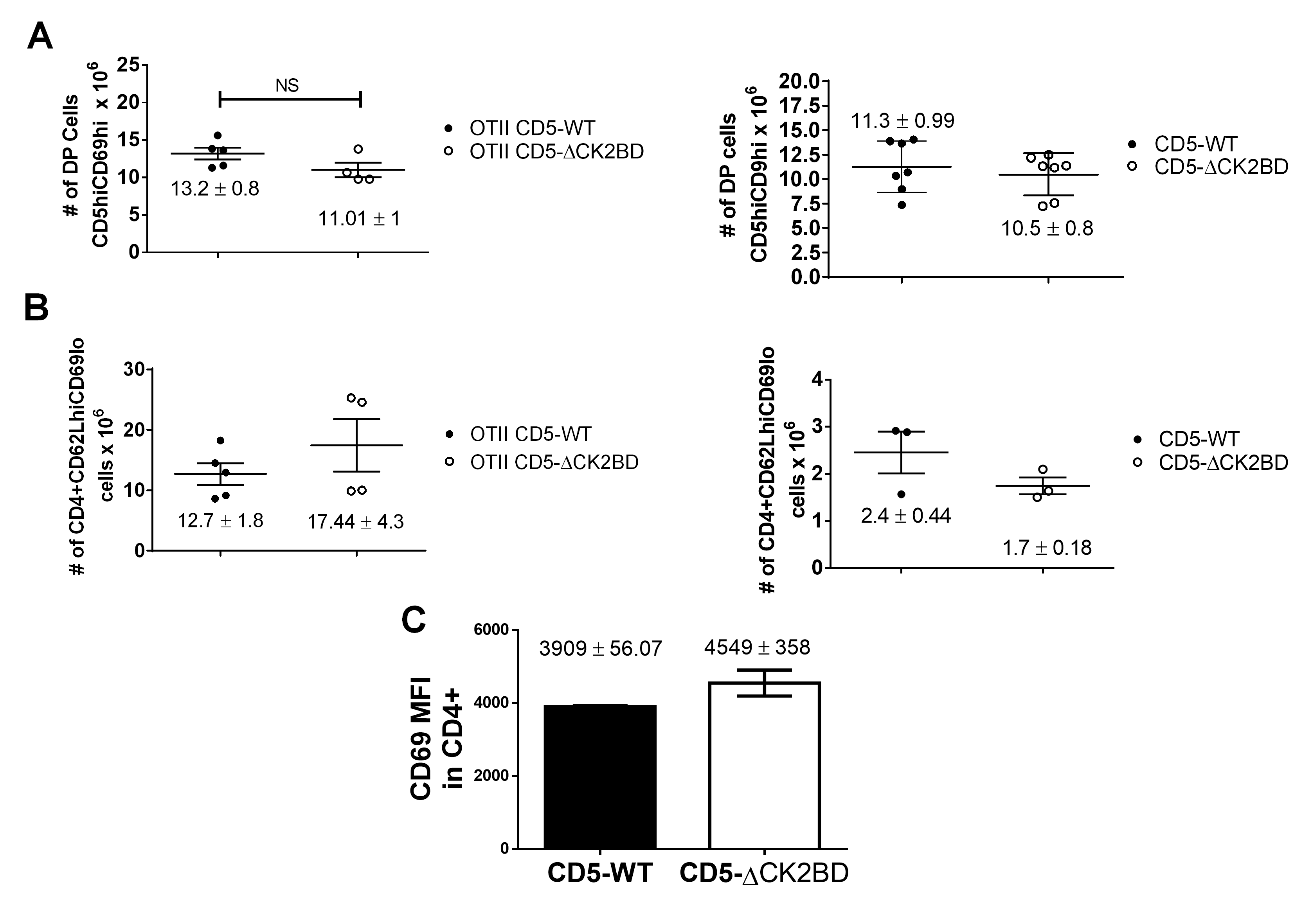

Supplement: S3 Fig — (A) Scatter plot (right panel, CD5-WT and CD5-ΔCK2BD OTII, left panel CD5-WT and CD5-ΔCK2BD non-Tg) representing total cell numbers from Fig 2A and 2B (CD5hiCD69hi cells). (B) Scatter plot (right panel, CD5-WT and CD5-ΔCK2BD OTII, left panel CD5-WT and CD5-ΔCK2BD non-Tg) representing total cell numbers from Fig 2C and 2D. (CD4+CD62LhiCD69lo). Each dot represents an independent mouse. (C) Graph representation of CD69 levels within CD4+SP cells in CD5-WT and CD5-ΔCK2BD non-Tg mice. Data representative of at least 3 independent experiments (n = 3–5 mice). Numbers are presented as mean± SEM. *p<0.05 **p<0.01. NS represents no statistical significance, unpaired two-tailed Student-t test. (TIF) [file pone.0168155.s003.tif]

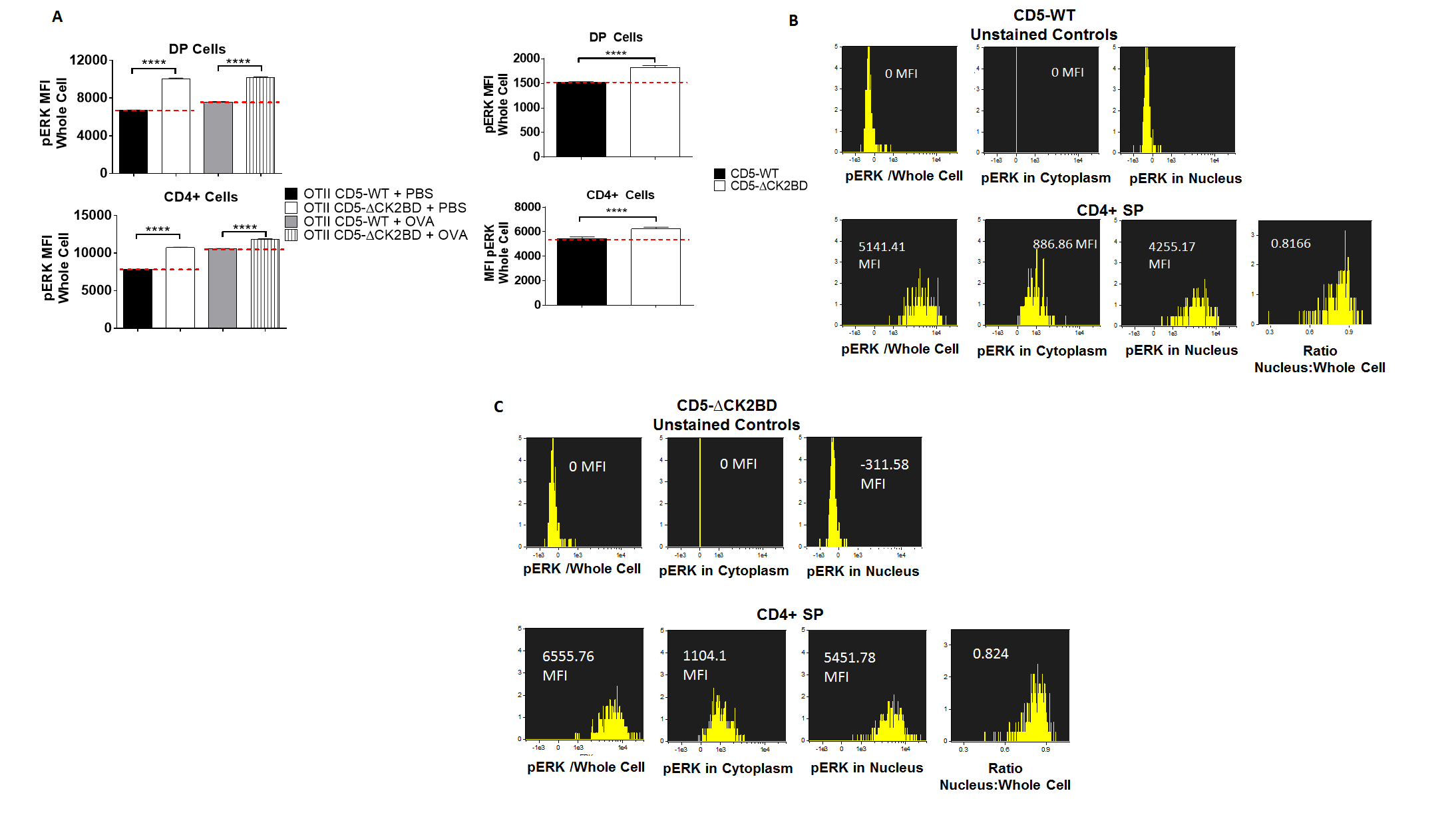

Supplement: S4 Fig — (A) Graphs representing total pERK levels detected by Imaging Flow Cytometry in CD4+SP and DP cells obtained from CD5-WT OTII and CD5-ΔCK2BD OTII TCR-Tg under basal conditions or after OVA stimulation (left panels), and from CD5-WT and CD5-ΔCK2BD non-Tg mice under basal conditions (right panels). (B) Histograms from a representative experiment, showing pERK levels in whole cell, cytoplasm and nucleus of CD4+SP cells obtained from CD5-WT (B) and CD5-ΔCK2BD non-Tg mice, under basal conditions. Nucleus:Whole cell ratio are also represented. (B and C). Data represent 900–1500 images of 2–3 independent experiments (n = 2–3 mice) ****p<0.0001, unpaired two-tailed Student-t test. (TIF) [file pone.0168155.s004.tif]
